# The Timing of T Cell Priming and Cycling

**DOI:** 10.3389/fimmu.2015.00563

**Published:** 2015-11-05

**Authors:** Reinhard Obst

**Affiliations:** ^1^Institute for Immunology, Ludwig-Maximilians-University Munich, Munich, Germany

**Keywords:** clonal selection, T cells, primary immune response, expansion, differentiation, cell cycle

## Abstract

The proliferation of specific lymphocytes is the central tenet of the clonal selection paradigm. Antigen recognition by T cells triggers a series of events that produces expanded clones of differentiated effector cells. TCR signaling events are detectable within seconds and minutes and are likely to continue for hours and days *in vivo*. Here, I review the work done on the importance of TCR signals in the later part of the expansion phase of the primary T cell response, primarily regarding the regulation of the cell cycle in CD4^+^ and CD8^+^ cells. The results suggest a degree of programing by early signals for effector differentiation, particularly in the CD8^+^ T cell compartment, with optimal expansion supported by persistent antigen presentation later on. Differences to CD4^+^ T cell expansion and new avenues toward a molecular understanding of cell cycle regulation in lymphocytes are discussed.

## Introduction

The priming of naive T cells, i.e., their activation following a primary recognition of specific peptide–MHC complexes, consists of a series of biophysical, biochemical, genetic, and proliferative events that lead to populations of expanded clones of differentiated effector cells, some of which have the potential to become long-lived memory cells. This clonal lymphocyte expansion is at the center of the still ruling clonal selection paradigm of adaptive immunity by Talmage, Lederberg (1, 2), and Burnet who hypothesized that lymphocyte “proliferation will be initiated of all those clones whose reactive sites correspond to the antigenic determinants on the antigen used” (3). Soon after its proposal, it was observed that indeed a proportion of small parental lymphocytes transferred into neonatal F1 hosts enlarge, become “pyroninophilic,” i.e., express large amounts of mostly ribosomal RNA, and incorporate tritiated thymidine, i.e., proliferate, before the recipients succumb to graft-versus-host disease 2–3 weeks later (4, 5). Quickly, such results were translated to *in vitro* proliferation assays for the detection of immunoresponsive cells among splenocytes from immunized animals (6) or human allo-reactive lymphocytes (7). The identification of the TCR proteins and genes and how to trigger T cell responses by monoclonal antibodies and second messenger agonists set the stage for closer analyses of the molecular events initiating proliferation (8–10) for which the mutagenesis of T cell tumor cell lines has been an especially fruitful approach (11). The adoptive transfer of TCR-transgenic T cells and the use of tracking dyes visualized cell populations expand and contract *in vivo* (12, 13). The detection of endogenous T cells of defined specificities by restimulation or tetramer assays also confirmed that the key driver of adaptive immunity is antigen (14, 15).

Research on the biophysics of TCR-peptide/MHC interaction (16, 17), the biochemistry of signal transducers (17, 18), transcription (19), and proliferation has naturally focused on different time frames of seconds, minutes, hours, and days, respectively, while an overall integration of these events, as noted recently, is still missing (20). The TCR signals convert a small metabolically inactive resting cell into an expanded group of descendants with newly acquired migratory and effector functions. While the dominant function of CD8^+^ T cells is the deletion of cells infected by intracellular pathogens like viruses and bacteria, their CD4^+^ counterparts differentiate following microenvironmental cues into discrete lineages profiled by cytokines expressed (21, 22). Over the following weeks, T cell numbers decline and a residual population survives as memory cells. Important in this context is that differentiation and proliferation coincide but are molecularly not necessarily coupled (23).

Here, we discuss the effects and requirements of TCR signaling for T cell proliferation in the primary response: What aspects of T cell differentiation follow analog versus digital logics of signal processing? Is T cell proliferation programed early on or is it maintained by continued antigen triggers? These questions have been addressed in a variety of experimental systems. The data further our understanding of the nature of immune responses to complex pathogens and our capability to design more immunogenic T cell vaccines (24).

## Lessons Learned from Intravital Imaging

Experiments using intravital 2-photon microscopy showed that in the steady state, a dendritic cell (DC) interacts with 500–5000 T cells per hour, which migrate within lymph nodes with a velocity of 10–12 μm per min, thereby scanning uncounted peptide/MHC complexes (25–27). Interestingly, CD4^+^ and CD8^+^ T cells employ different strategies of surveillance: The interaction times of CD4^+^ T cells with DCs depend on MHC class II (MHC-II) molecules while CD8^+^ T cells traverse a LN slower and regardless of self-peptide/MHC-I complexes. They scan 160–200 and 300 DCs per hour and, thus, stay in a lymph node approximately for half a day and a day, respectively (28). Considering natural precursor frequencies, it has been assessed that at least 85 antigen-presenting DCs per lymph node are necessary to initiate a CD4^+^ T cell response (29). When confronted with a DC presenting antigen, specific T cells stop migrating and stay in touch with an individual DC for around a day (30–33). Within this period, the T cells undergo changes classically summarized as “blasting”: They increase in size, double their protein contents, increase their total RNA contents 30-fold, induce the expression of around 1300 mRNAs, and change their metabolism before proliferation ensues 24 h later (34–40).The stable interaction with one DC can be preceded by a phase of transient interactions with several APCs, the length of which is inversely correlated with APC density and antigen dose (41). Interestingly, it has been shown that, for CD8^+^ T cells, the phase of stable pairing with one DC is not necessarily required for expansion of effector clones, while memory differentiation is affected. These findings indicated that the memory potential of CD8^+^ T cells can be programed within the first 24 h of priming (36). The data also supported the relevance of observations that T cells can “memorize” sequential sub-threshold interactions with different APCs and accumulate such signals over time, perhaps via AP1 or NFAT (42–46).

## Counting Precursors and Effectors to Assess the Level of Expansion

The end result of priming is a population of expanded clones, the numerology of which has recently been assessed with great precision. It turned out that the precursor frequency of specific cells in the naive repertoire is a critical parameter for the magnitude of a T cell response. Even for “strong” antigens with unusually high precursor frequencies like alloantigens, estimates using classical techniques like limiting dilution analysis have been notoriously variable by several orders of magnitude (47), before the actual frequency of around 10% could be clarified with cell-tracking dyes *in vivo* (48, 49). However, the much lower precursor frequencies to standard antigens could not be determined until peptide/MHC tetramers were employed for enriching the naive precursors from unimmunized animals. This technology is likely geared toward the detection of high-affinity T cells and will evolve further (50–53). For CD8^+^ T cells, precursor frequencies were found between 1 and 100 cells per 10^6^ cells, while the numbers were three to five times lower for CD4^+^ T cells in both mouse and man (54, 55), confirming previous findings based on single-cell transfers and comparisons with expanded cells derived from the endogenous repertoire (56). Such numbers are important as they can partially predict the T cells’ clonal burst size and an individual’s immunological potential, though other mechanisms like antigen presentation efficiency, peptide/MHC stability, kind of APC, inflammation, niche issues, and self-reactivity likely contribute (57–65). Importantly, the traficking and microfluidics of the secondary lymphoid organs are arranged to make priming an efficient process so that all existing rare precursors are recruited into an immune response rapidly and efficiently (29, 33, 66). Tetramer and restimulation assays have allowed for a determination of the T cells’ clonal burst size and, thus, the determination of their proliferative capacity.

In responses to several epitopes derived from lymphocytic choriomeningitis virus (LCMV), an acute viral infection that triggers exceptionally strong T cell responses, CD8^+^ T cells divide about 15 times (14, 15) and 16–19 times in response to the Gram-positive intracellular bacterium *Listeria monocytogenes* (67). More recently, two studies on clones derived from individually labeled CD8^+^ T cells responding to *Listeria* showed, on average, 15 divisions, but also a wide, and mysterious, variation of 10–20 divisions that was independent of TCR specificity (58, 59). By contrast, tetramer studies determined the division numbers of CD4^+^ T cells to 7 on average, with different specificities falling between 4 and 10 divisions (54, 55, 68). This difference between CD4^+^ and CD8^+^ T cells confirms earlier studies in LCMV, vaccinia and Sendai virus infections where CD8^+^ T cells expand to a much greater extent than their CD4^+^ counterparts (69–71) and an intrinsic difference between the cell types, regarding their proliferative potential, had been suggested early on (72, 73).

## Autopilot Experiments: CD8s

Early immunizations of TCR-transgenic *cd28*-deleted animals had suggested that the costimulatory requirements of CD8^+^ T cell priming can be overcome by prolonged antigen presentation (74). However, since it was quickly seen that TCR-transgenic animals are no models of T cell expansion (75, 76), more physiological systems were developed. To investigate the role of antigen persistence in the process of T cell priming the TCR signal has to be interrupted experimentally. The first approach to do this was to prime naive CD4^+^ T cells in plates coated with peptide/MHC complexes and transfer the cells to uncoated wells at different times, with the effects of costimulation, APC, and responder cell types assessed as well. The authors concluded that sustained TCR signaling is the key parameter for the priming of naive CD4^+^ T cells (77).

These results provoked work on the antigen dependence of CD8^+^ T cell expansion that projected a different picture. Naive P14 TCR-transgenic T cells primed for 24 h continued their proliferation in antigen-free cultures and upon transfer into antigen-free recipients (78). Experiments with OT-1 T cells primed with fibroblast transfectants or antibodies showed that a priming period as short as 2 or 2.5 h was sufficient for the T cells to continue their divisions over the following days *in vitro* (79, 80). In a follow-up study, however, it was shown that a 20-h period of priming was necessary for the quantitative maintenance of the cells upon transfer into recipient animals, indicating that proliferation *in vitro* can reflect an incomplete level of activation that leaves the cells ill-equipped for survival in secondary lymphoid organs (81). Not entirely consistent was the later observation that the 4-h-primed cells survive sufficiently to protect recipients against an OVA-expressing tumor (82).

The difference between the 2001 and 2003 studies by the Schoenberger group done *in vitro* and partially *in vivo* (79, 81) was later shown to be based on a factor working *in trans* between T cells cultured at high densities, namely IL-2 (83). Importantly, the merely accessory role of IL-2 for primary T cell expansion *in vivo* has been demonstrated in several infectious, transplantation, and vaccination models (84). For antigen-independent proliferation of CD8^+^ T cells, IL-2 has a role *in vitro*, but not *in vivo* (80, 85). Though IL-2 has been used for culturing T cell lines and clones in the laboratory for decades, *in vivo* it mostly affects the maintenance of CD25^+^ regulatory T cells (86–88), which are the only T cells where IL-2/STAT5 signaling can be detected following immunization (89), and the programing of an efficient secondary response (90, 91). However, the contribution of IL-2 signals to T cell expansion *in vivo* is not 0 and its actions may transmit signals from regulatory T cells and the inflammatory microenvironment (57, 92–94).

The results of the 2001 “autopilot” publications had been foreshadowed by experiments using the timed application of ampicillin to remove intracellular antigen-delivering *L. monocytogenes* bacteria from infected animals. The expansion of CD8^+^ T cells specific for two *Listeria*-derived epitopes continued, despite the efficient removal of live bacteria 24 h p.i. (95). In hindsight, one may ask what the half-life of residual antigen presentation, which has been reported in several infection models since, might have been. However, the swift disappearance of T cell antigen from *Listeria*-infected animals within a day after antibiotic treatment was shown later by T cell transfers and *in vitro* assays and supported the initial conclusion that CD8^+^ T cell proliferation is programed *in vivo* within the initial 24 h of priming (96, 97). The observation that T cell contraction is programed as well strengthened this view (98, 99).

These observations are widely cited and considered seminal (100–103). The behavior of CD8^+^ T cells to execute a program set in place within the first day of priming has been illustratively summarized as being “on autopilot” or “programed” (100, 102). However, there are a significant number of publications that do not fit the scheme. Curtsinger et al. showed that adoptively transferred OT-1 T cells primed *in vitro* 16 h earlier did not proliferate and accumulate in antigen-free hosts compared to previously vaccinated ones (104). It is possible that in these experiments the programing threshold at 24 h had not yet been reached. The paper also showed that inflammatory stimuli can affect CD8^+^ T cell expansion, in agreement with experiments using P14 TCR-transgenic cells whose cognate antigen D^b^/LCMV-GP_33–41_ has a short half-life *in vivo*. Comparing animals immunized with peptide, virus-like particles, and live virus, CD8^+^ T cell expansion correlated with antigen persistence, clearly arguing against an “autopilot” model (105). In the *Listeria* model, it was reported that ampicillin treatment between 24 and 60 h p.i. affected both endogenous CD4^+^ and CD8^+^ T cell expansion when read out in a peptide restimulation assay (106). The main difference to the earlier reports from four different laboratories was that they had used adoptive transfers of TCR-transgenic T cells, allowing for a clearly defined starting point of the expansion phase, and had visualized the proliferating T cells directly by fluorescent tracking dyes (78, 80, 85, 96, 97).

In immunizations with irradiated *Plasmodium yoelii* sporozoites, it was shown that a T cell antigen derived from them can persist for months. Using consecutive transfers of TCR-transgenic T cells specific for *P. yoelii* between recipients immunized with *P. yoelii* or *Plasmodium falciparum*, it was shown that antigen presentation beyond day 4 significantly contributes to the number of memory cells detectable a month later, implying that it supported a better primary response and revising an earlier interpretation (107, 108).

Another approach to limit the time of antigen exposure is the delayed T cell transfer of naive T cells into recipients infected days earlier. This procedure necessarily varies both parameters, dose and time, of priming antigen. In addition, the transferred cells face a rising competition of endogenous effector cells (109). All these parameters are likely to contribute to the result that the CD8^+^ T cells transferred at the peak of the response to vaccinia virus on day 7 exhibit overall inefficient priming with lower levels of division, survival, and memory differentiation (110).

The ablation of DCs from CD11c-diphtheria toxin receptor (DTR)/green fluorescent protein (GFP) transgenic animals is another way to interrupt the interactions between T cells and APCs (111, 112). Such animals express a fusion protein of the DTR and GFP on the surface of DCs. Mice are naturally resistant to diphtheria toxin as they lack a receptor for this toxin, so that only transgene-expressing DCs and some macrophages are depleted (113). Since the expression of such conventional transgenes is, like many others, mosaic, the depletion of DCs from such animals is incomplete. In addition, the fact that the depletion of large numbers of DCs in transgenics directly modifies lymphocyte homing to lymph nodes via high endothelial venules is an unintended consequence (114). Thus, the procedure employed by Prlic et al., who sorted DTR/GFP^+^ DCs, transferred them after peptide loading, and depleted them at different time points of a T cell response, avoided these potential pitfalls. Responding TCR-transgenic cells accumulated linearly with the delay of the depletion, while their functionality in primary and secondary responses remained unchanged (115). Similar results were presented in an elegant study that terminated TCR signals by turning off a tetracycline-inducible *lck* gene during the expansion phase of endogenous cells responding to vaccinia virus (116). Also, the expansion of cells primed *in vitro* was significantly supported only in recipients that underwent an infection with the cognate antigen (117). Thus, these three datasets supported the interpretation that the differentiation of CD8^+^ T cells is programed within the initial 24 h of priming, but not their quantitative accumulation.

Blocking antigen presentation *in vivo* with peptide/MHC-specific antibodies has been difficult as only few reagents with a sufficiently high affinity exist. Blair et al. used antibodies specific for K^b^/Ova_257–264_ and A^b^/Eα_52–68_ to block the priming of TCR-transgenic T cells with the respective specificities following systemic infections with modified VSV. The results showed that both CD4^+^ and CD8^+^ T cell responses were affected. Though the antibodies blocked T cell expansion only incompletely, the results argue against the autopilot mode of T cell expansion (118).

These discrepancies cannot easily be explained by experimental details and modes of infection. We are left with two groups of publications of about equal numbers, arguing for and against an antigen-independent phase of CD8^+^ T cell expansion. Perhaps one is left with the understanding that CD8^+^ T cell proliferation and differentiation is initially programed, but is not hardwired and can be modified by later TCR signals (21). Several more recent studies looking at the effects of viral antigen presented locally support this view.

In the first 2 days of a pulmonary influenza infection, alveolar DCs migrate to the draining lymph nodes for priming (119, 120). The remaining DCs and macrophages can be partially depleted by i.n. application of clodronate-containing liposomes. By using this technique, McGill et al. showed that CD8^+^ T cells expand in response to antigen presented by such APCs in the lung, suggesting a “two-hit model” of CD8^+^ T cell expansion (121, 122). The depletion of DCs from CD11c-DTR/GFP transgenics on day 6 p.i. also reduces the number of CD8^+^ effector cells in the lungs significantly (123). An additional role of antigen presentation following day 7 for the CD8^+^ memory cell functionality was shown by León et al. in the same system. This paper with insight showed that late antigen presentation depended on specific IgG and FcγR^+^ DCs, suggesting that immune complexes are cross-presented to CD8^+^ T cells late in the primary response and affect proper memory cell differentiation (124). A division of labor for effector and memory cell differentiation was also shown for two migratory DC types, one expressing CD103, the other CD11b, arguing for more nuanced T cell-APC interactions via costimulatory molecules, here CD24 (119). In the LCMV infection model, Kang et al. followed primed T cells at the peak of the response entering the CNS and found them synthesizing DNA, implying that they left the secondary lymphoid organs while actively cycling. In the CNS, the cells established long-lived interactions with local DCs and T cell transfers into MHC-I-negative recipients showed that it is local antigen presentation that supports additional divisions within the CNS (125). These data from infection models stress the role late antigen presentation has on CD8^+^ T cell proliferation.

## Autopilot Experiments: CD4s

The initial *in vitro* experiments by Iezzi et al., reporting the necessity of antigen persistence for several days, were done with CD4^+^ T cells exposed to peptide/MHC complexes for limited periods of time and then recultured in new dishes (77). These findings were reproduced in experiments with antigen-loaded APCs by interrupting the TCR signals with MHC-specific antibodies (126–128) and then *in vivo* in the *Listeria* model where the antigen removal by ampicillin affected the CD4^+^ T cell responses much more than those of the CD8^+^ T cells (96, 97). A careful study using a heterologous rechallenge extended these findings to secondary responses and established that CD4^+^ T memory cells do not acquire an “autopilot” phenotype (129). A direct demonstration of antigen dependence of the CD4^+^ T cell response was possible by using transgenic mice in which the presentation of an MHC-II restricted antigen by DCs could be controlled by doxycycline *in vivo*. Antigen withdrawal quickly arrested proliferation of adoptively transferred T cells (130). These findings were in agreement with results of Celli et al. who transferred antigen-presenting DCs consecutively and showed the enhanced expansion and effector differentiation of CD4^+^ T cells and, importantly, visualized the stable contacts between DCs and T cells at later stages in the priming process by intravital microscopy (131, 132). Also the residual antigen left behind following the resolution of an influenza infection and still visible to CD4^+^ T cells supports their memory differentiation (133).

A caveat to these studies was the high numbers of T cells transferred, a procedure that can affect the results (67, 134–137). However, the antigen dependence of CD4^+^ T cell expansion was also demonstrated by transferring small numbers of transgenic T cells (39, 138). An additional *in vivo* study looking at anti-HY responses of polyclonal T cells showed that antigen persistence is necessary for CD4^+^ T cells to expand and license DCs for proper CD8^+^ priming in a system relying entirely on natural precursor frequencies (139).

This work supported the model that CD4^+^ T cells require contact with APCs and TCR signals to keep the cell cycle going for several rounds. There are, nevertheless, a number of experiments, mostly done *in vitro*, that show antigen-independent proliferation of CD4^+^ T cells, either supported by cytokines (140) or not (141, 142). Interestingly, some of them reported a dampening effect of antigen presented to CD4^+^ T cells repeatedly, which drives the cells into exhaustion (143), a finding with clear correlates *in vivo* (144) and in man (77, 145).

## Comparing CD4s and CD8s

The few direct comparisons between CD4^+^ and CD8^+^ T cells that have been done mostly observe a higher degree of programing in the CD8^+^ compartment. This was found in experiments with *Listeria* removed by ampicillin (96, 97) and a more recent side-by-side comparison of *in vitro*-primed CD4^+^ and CD8^+^ T cells. Here, not just proliferation but also functionality and gene expression profiles supported the different consequences of interrupted TCR signals by the two subsets (39). These findings resonate with a number of observations that intrinsically differentiate the two subsets. First, CD4 has a higher affinity to the key signaling molecule lck than CD8 so that different proportions of coreceptors are bound by lck (146, 147). Second, it has been reported in several systems that CD4/CD8 lineage commitment in the thymus is regulated by the kinetics of the positively selecting signal (148–150). Third, naive CD4^+^ T cells traverse lymph nodes faster than CD8^+^ T cells and do so in an MHC-dependent way (28). Fourth, tissue-resident CD4^+^ T memory cells recirculate via lymph and blood while their CD8^+^ counterparts stay put in the tissues and persist there in the absence of antigen (151). Fifth, the different importance of programing for proliferation may explain the different numbers of divisions observed in the two subsets mentioned before (54). And sixth, the higher degree of plasticity of CD4^+^ T cells is reflected in the multiplicity of lineages they can differentiate into under the guidance of cytokines and transcription factors (22).

In addition, there are also cell-extrinsic differences between the two subsets: MHC-I molecules are expressed by all nucleated cells, but MHC-II by professional APCs only. The substrates of antigen presentation via MHC-I may be short-lived defective ribosomal products rather than comparably stable proteins for the MHC-II pathway (152). Thus, one might speculate that the aspect of programing of CD8^+^ T cells reflects the more transient antigen presentation by MHC-I (39). Upon activation, the stability of MHC-II molecules on DCs is tightly regulated (153, 154) via ubiquitination (155, 156), presumably by MARCH family members (157, 158), while that of MHC-I molecules is not (39, 153). There is also increasing evidence that the two T cell subsets are primed by different DCs. Antigen targeting with the two antibodies DEC205 and 33D1 revealed that CD8^+^ T cells are primed by CD205^+^CD8^+^ DCs, while CD4^+^ T cells are engaged best by DCIR2^+^CD8^−^ DCs (159). These pivotal observations were recently confirmed by genetic means: the two APC subsets are also differentiated by the transcription factors IRF4 and IRF8, respectively (160). Intravital 2-photon microscopy of animals systemically immunized with a non-replicative vaccinia virus has shown directly that the initial priming of CD4^+^ and CD8^+^ T cells occurs at spatially separate sites in the lymph node by different DCs. In later phases of the response, CD4^+^ and CD8^+^ T cells were seen clustered around the same XCR1^+^ DCs where T cell help is transmitted, which is evidenced by the finding that CD8^+^ T cells primed in XCR1^+^-DC-depleted animals display a “helpless” phenotype (161). A similar division of labor among priming DCs has been shown for local HSV infections where antigen presentation also involves migratory DCs (162). A key question in the future will be how this spatial separation is accomplished and what purpose it might serve.

## Outlook: New Approaches to Interrupt TCR Signals and to Visualize the Cell Cycle of T Cells

The shortcomings of the techniques used to study the role of antigen for T cell proliferation so far has recently motivated new approaches. To quickly and specifically interrupt TCR signals at will have been notoriously difficult, especially *in vivo* [e.g., Ref. (39)]. Art Weiss’ laboratory has worked toward this goal by developing a variant of the indispensible signal transducer ZAP70 whose kinase domain is sensitive to the ATP analog 3-MB-PP1 (163, 164). The combination with a novel Nur77-GFP reporter (165) allowed for the visualization of TCR signals and their proliferative consequences with ZAP70 abruptly turned off at different time points following stimulation *in vitro*. The experiments demonstrated a clear temporal threshold of 24 h for both CD4^+^ and CD8^+^ T cells to commit to proliferation that then commences, mostly driven by IL-2. Both subsets also continued to divide several times following the termination of ZAP70 signaling (166). Experiments using such techniques *in vivo* to follow immune responses with signaling terminated abruptly will certainly open new windows in the future.

The fact that tracking dyes cannot be resolved by flow cytometry beyond the eighth division, before the expansion of CD8^+^ T cells is finished, calls for other ways to document the proliferative status of cell populations, especially late in the response. Classical and novel DNA dyes visualize the cell cycle status of heterogeneous cell populations (167, 168) and new antibodies against cell cycle components are available, at least for human cells (169). The nucleoside analog EdU whose detection is based on click chemistry will certainly assist future research on cell cycle control in lymphocytes (170). Particularly, the consecutive injections of BrdU and EdU allow the precise labeling of cells in S phase *in vivo* even of cells with a complex history: Nussenzweig and colleagues showed by this technique that follicular helper T cells accelerate the cell cycle of germinal center B cells (171, 172). The doxycycline-induced expression of a very stable GFP–H2B fusion protein that then gets diluted upon division has been used to visualize the proliferative history of cells without liberating them from their microenvironment, like hematopoietic stem cells and germinal center B cells (171, 173). Another novel approach is the use of a double transgenic mouse that expresses two different fluorescent dyes under cycle-dependent promoters to differentiate G0/G1 from S/G2/M phases (93, 172, 174–176). *In vitro*, tracking family trees of divided cells by time lapse microscopy showed that the division program, called its division destiny, is programed prior to the first division and passed on for several generations (93). Following CD8^+^ TCR-transgenic cells through a primary response to influenza, Kinjyo et al. observed that the cells divide at least eight times in a homogeneous and fast manner. At the peak of the response at day 7, however, a small CD62L^hi^ subpopulation emerges that slows down its cell cycle and express gene sets similar to memory cells. By following division trees *in vitro*, the authors show that phenotype and cell cycle duration are inherited to daughter cells, suggesting a cell-intrinsic program to diversify the proliferative activity in the priming phase of CD8^+^ T cells along the split between short-lived and memory-precursor effector cells (176). These data indicate a direction for future research.

Most of the genes analyzed so far are general cell cycle genes active in many cell types: cyclin D3 (177), p27*^*kip1*^* (178, 179), CDK5 (180), CDK6 (181), Bcl11b (182), FoxM1 (183), myc (23), and Geminin (184). However, the first exception is CTP synthase 1 that exclusively affects lymphocyte proliferation in individuals lacking a functional allele (185). Nevertheless, the molecular regulation of the cell cycle in lymphocytes, and, thus, the core of the clonal selection paradigm, is still a black box. Since the cell cycle of lymphocytes is four to five times faster than that of, e.g., Hela cells, one would assume a specific machinery or cell-type-specific components that run or control proliferation in the adaptive arm of the immune system.

## Conflict of Interest Statement

The author declares that the research was conducted in the absence of any commercial or financial relationships that could be construed as a potential conflict of interest.
